# Cascade CRISPR/cas Enables More Sensitive Detection of *Toxoplasma gondii* and *Listeria monocytogenes* than Single CRISPR/cas

**DOI:** 10.3390/microorganisms13081896

**Published:** 2025-08-14

**Authors:** Dawei Chen, Min Sun, Bingbing Li, Jian Ma, Qinjun Zhang, Wanli Yin, Jie Li, Mingyue Wei, Liang Liu, Pengfei Yang, Yujuan Shen

**Affiliations:** 1 NHC Key Laboratory on Parasite and Vector Biology, National Institute of Parasitic Diseases, Chinese Center for Disease Control and Prevention, Chinese Center for Tropical Diseases Research, National Key Laboratory of Intelligent Tracking and Forecasting for Infectious Diseases, WHO Collaborating Center for Tropical Diseases, Shanghai 200025, China; chendawei19911104@163.com (D.C.); sunmsunny@163.com (M.S.); 2 Huai’an Center for Disease Control and Prevention, Jiangsu Provincial Regional Public Health Center, Huai’an 223003, China; hacdclibingbing@163.com (B.L.); hacdcmj.888@163.com (J.M.); 13815704893@163.com (Q.Z.); hacdcyingwanli@163.com (W.Y.); hacdclijie@163.com (J.L.); hacdcweimingyue@163.com (M.W.); hacdcliuliang@163.com (L.L.);

**Keywords:** amplification-free CRISPR detection, foodborne pathogen, Cascade-CRISPR, salt concentration

## Abstract

Foodborne pathogens represent a class of pathogenic microorganisms capable of causing food poisoning or serving as foodborne vectors, constituting a major source of food safety concerns. With increasing demands for rapid diagnostics, conventional culture-based methods and PCR assays face limitations due to prolonged turnaround times and specialized facility requirements. While CRISPR-based detection has emerged as a promising rapid diagnostic platform, its inherent inability to detect low-abundance targets necessitates coupling with isothermal amplification, thereby increasing operational complexity. In this study, we preliminarily developed a novel amplification-free Cascade-CRISPR detection system utilizing a hairpin DNA amplifier. This method achieves detection sensitivity as low as 10 fM (82 parasites/μL) for DNA targets within 30 min without requiring pre-amplification, with background signal suppression achieved through optimized NaCl concentration. Validation using artificially contaminated food samples demonstrated the platform’s robust performance for both *Toxoplasma gondii* (*T. gondii*) and *Listeria monocytogenes* (*L. monocytogenes*) detection, confirming broad applicability. In summary, this study preliminarily establishes an amplification-free Cascade-CRISPR detection platform that achieves high sensitivity and rapid turnaround, demonstrating strong potential for on-site screening of foodborne pathogens.

## 1. Introduction

Molecular diagnostic tools play a crucial role in pathogen identification and controlling the transmission of pathogenic bacteria. Although polymerase chain reaction (PCR) technology has long been considered the gold standard for molecular diagnosis, it requires specialized instrumentation, trained personnel, and controlled laboratory environments. Consequently, significant efforts have been devoted to developing more accessible and simplified molecular diagnostic technologies. CRISPR technology was initially developed for gene editing. Subsequent studies revealed that Cas13, Cas12, Cas14, and Cas9 proteins also possess trans-cleavage activity [1,2,3,4]. According to the trans-cleavage activity of Cas proteins, a series of detection platforms have been developed, including HOMES, DETECTOR, and SHERLOCK. CRISPR-based detection technologies have attracted significant attention due to their high sensitivity, specificity, and cost-effectiveness. When combined with lateral flow assay (LFA) technology, CRISPR-based detection becomes more portable, enabling nucleic acid testing without the need for expensive PCR instruments [5,6,7].

Although CRISPR-based detection offers numerous advantages, it still requires integration with isothermal amplification or PCR for low-abundance targets [8,9,10]. However, compatibility issues exist between CRISPR detection and amplification systems (e.g., isothermal amplification or PCR). Consequently, the initial approach involved conducting amplification and CRISPR detection in separate steps, where amplification preceded detection [11,12]. To address this limitation, a one-pot assay was subsequently developed, combining isothermal amplification and CRISPR detection in a single reaction tube [13,14]. These technological advancements have established CRISPR-based detection as a next-generation molecular diagnostic tool.

While the integration with isothermal amplification enables the detection of low-abundance targets, it concomitantly increases costs and operational complexity, thereby limiting the broader application of CRISPR-based diagnostics. Consequently, significant efforts have been devoted to developing amplification-free CRISPR detection technologies. For instance, Sha et al. [15] achieved femtomolar (fM) sensitivity by conjugating Cas13 and Cas14 proteins through hybrid DNA hairpins. Similarly, Shi et al. [16] engineered a split CRISPR guide RNA (scgRNA) with dual functionality: Upon cleavage, it generates both a fluorescent signal and a novel target DNA recognized by the T2 complex. Subsequent T2 complex activation induces further scgRNA cleavage, establishing a positive feedback loop that exponentially amplifies the fluorescent signal. Additionally, Lim et al. [17] attained attomolar (aM) detection sensitivity using biotin-labeled hairpin DNA as a signal amplifier. Despite these advancements, current amplification-free CRISPR detection systems face challenges, including a complex signal amplifier, high background signals, and a predominant reliance on LbCas12a protein, with limited reports on alternative variants such as AsCas12a and FnCas12a.

Foodborne pathogens are biological pathogenic agents that enter the human body through contaminated food or water, capable of causing infections, intoxication, or other health hazards. Representative examples include *Toxoplasma gondii* and *Listeria monocytogenes*. The transmission of *T. gondii* can be categorized into horizontal and vertical transmission. Horizontal transmission occurs via consumption of raw or undercooked meat contaminated with *T. gondii* or ingestion of vegetables and water contaminated with oocysts, while vertical transmission refers to the transplacental passage of tachyzoites from mother to fetus [18,19]. Immunocompromised individuals infected with this parasite may develop life-threatening parasitic diseases, typically manifesting as encephalitis, pneumonia, myocarditis, etc. [20]. *Listeria monocytogenes* (*L. monocytogenes*) is an intracellular pathogen that can cause spontaneous abortion, neonatal sepsis, and meningoencephalitis [21,22]. Unlike other foodborne pathogens primarily affecting the gastrointestinal tract, *L. monocytogenes* can cross the blood–brain barrier and placental barrier, exacerbating disease severity [21]. To safeguard public health and food safety, rapid and straightforward methods for detecting foodborne pathogens are imperative.

This study preliminarily established an amplification-free CRISPR detection platform by utilizing hairpin DNA as a signal amplifier in conjunction with AsCas12a protein. Through optimization of buffer composition and hairpin DNA configuration, femtomolar-level DNA detection sensitivity was achieved. Notably, we demonstrated that AsCas12a outperformed the commonly used LbCas12a protein in this system, and that high-salt conditions effectively suppressed background signals generated by hairpin DNA. The method’s sensitivity and specificity were rigorously evaluated using *T*. *gondii* and *L*. *monocytogenes* as model targets, followed by validation with real samples. Collectively, this work presents a novel amplification-free CRISPR-based detection approach with high sensitivity and specificity.

## 2. Materials and Methods

### 2.1. Preparation of Proteins and Nucleic Acids

The CRISPR/Cas12a proteins, including AsCas12a (Cpf1) nuclease (32104) and LbCas12a (Cpf1) nuclease (32108), were procured from Tolobio Technology and diluted according to experimental requirements prior to use. The *Toxoplasma gondii* B1 gene fragment was synthesized by Sangon Biotech (Shanghai, China), dissolved, and stored at −20 °C for subsequent experiments. The crRNA sequences were designed using the online tool available at https://ezassay.com/primer (accessed on 25 May 2024). The hairpin DNA was designed based on the report by Jae Hoon Jeung et al. [23], while the reporter probe was selected with reference to the study by Li et al. [24]. All oligonucleotides, including crRNA, hairpin DNA, and reporter probes, were synthesized by Sangon Biotech (Shanghai). The synthesized hairpin DNA was dissolved in TE buffer (pH 8.0) and diluted to a final concentration of 10 μM. Subsequently, gradient annealing was performed to ensure proper secondary structure formation before use. Oligonucleotide sequences utilized in this study can be found in Table S1.

### 2.2. Polyacrylamide Gel Electrophoresis (PAGE) Analysis

The polyacrylamide gel electrophoresis (PAGE) kit was purchased from Sangon Biotech (Shanghai, China, C631101-0100). A 20 μL reaction mixture was prepared as follows: 1 μL of 1 μM Cas12a protein, 1 μL of 2 μM crRNA, 1 μL of 10 ng/μL target DNA, 2 μL of 10× NEB buffer 3.1, 1 μL of 10 μM hairpin DNA (HP-DNA), 14 μL of deionized water. The reaction was incubated at 37 °C for 60 min to allow Cas12a-mediated cleavage of the hairpin DNA loop region, converting it into linear DNA. A 15% polyacrylamide gel was prepared with the following components: 6 mL of 30% acrylamide/bis-acrylamide solution, 3.6 mL of distilled water, 2.4 mL of 5× Tris-Borate-EDTA (TBE) buffer, 200 μL of 10% ammonium persulfate (APS), 10 μL of TEMED (N,N,N’,N’-tetramethylethylenediamine). The gel was polymerized at room temperature before electrophoresis. Electrophoresis conditions: running buffer: 1× TBE, voltage: 100 V, duration: 75 min. After electrophoresis, the gel was stained with GelRed Nucleic Acid Stain for 20 min and visualized using the GelDoc XR Imaging System (Bio-Rad, Hercules, CA, USA).

### 2.3. Validation of Hairpin DNA Effects on Cas12a Trans-Cleavage Activity

The Cas12a/crRNA complex was prepared by mixing 1 μL of 1 μM Cas12a protein and 1 μL of 2 μM crRNA. The mixture was incubated at 37 °C for 10 min to facilitate the formation of the Cas12a/crRNA ribonucleoprotein (RNP) complex.

Evaluation of Inhibitory Effects: The inhibitory activity was assessed using a 20 μL CRISPR/Cas12a reaction system containing 1 μL RNP2 (pre-assembled Cas12a/crRNA complex 2), 1 μL 10 μM hairpin DNA, 2 μL 10× NEB buffer 3.1, 1 μL 10 μM fluorescent reporter probe, and 15 μL nuclease-free water. The reaction mixture was incubated at 35 °C for 60 min, with real-time fluorescence measurements recorded using QuantStudio5.

Evaluation of Activatory Effects: The activatory effects were assessed using a 20 μL primary reaction containing 1 μL RNP1 (pre-assembled Cas12a/crRNA complex 1), 1 μL 10 ng/μL target DNA, 2 μL 10× NEB buffer 3.1, 1 μL 10 μM hairpin DNA, and 15 μL nuclease-free water. After 60 min incubation at 37 °C, the reaction was heat-inactivated at 85 °C for 10 min to denature Cas12a activity, followed by cooling to room temperature. Then, 1 μL RNP2 and 1 μL 10 μM ssDNA reporter were added to the mixture. The reaction proceeded at 37 °C for 40 min with continuous fluorescence monitoring.

### 2.4. Single Cas12a Assay

The Cas12a/crRNA complex was prepared by mixing 1 μL of 1 μM Cas12a protein and 1 μL of 2 μM crRNA. The mixture was incubated at 37 °C for 10 min to facilitate the formation of the Cas12a/crRNA ribonucleoprotein complex. The reaction scale could be proportionally increased based on experimental requirements. For a single Cas12a reaction, a 20 μL reaction mixture was prepared as follows: 1 μL RNP, 2 μL of 10× NEB buffer 3.1, 2 μL target DNA, 1 μL 10μM ssDNA reporter, 14 μL of deionized water. The reaction was incubated at 37 °C for 60 min in a real-time fluorescence PCR system (QuantStudio5). Fluorescence signals (FAM) were recorded at 30 s intervals to monitor Cas12a-mediated trans-cleavage activity.

### 2.5. Cascade-CRISPR Assay

To simplify the workflow and minimize aerosol contamination, we integrated the target recognition and signal amplification steps into a single PCR tube. A 20 μL reaction mixture was prepared as follows: 1 μL RNP1 (pre-assembled Cas12a/crRNA complex 1), 1 μL RNP2 (pre-assembled Cas12a/crRNA complex 2), 2 μL target DNA, 2 μL 10× NEB buffer 3.1, 2 μL 0.5 μM hairpin DNA (hpDNA, substrate for signal amplification), 11 μL nuclease-free water, 1 μL 10 μM fluorescent reporter probe (FAM-ssDNA-BHQ1) on the inner surface of the PCR tube cap. After gently sealing the PCR tube, it was incubated at 30 °C for 20 min to allow target recognition and signal amplification. Then, the tube was centrifuged and mixed (8000 rpm,15 s). The reaction was incubated at 30 °C for 60 min while monitoring real-time fluorescence (FAM channel) at 30 s intervals using a QuantStudio5 (Thermo Fisher Scientific, Shanghai, China) qPCR instrument.

### 2.6. Evaluation of Reaction System Specificity and Sensitivity

To assess the specificity of the established detection system, genomic DNA from four distinct parasites (*Toxoplasma gondii, Clonorchis sinensis*, *Cryptosporidium parvum*, and *Babesia*) as well as genomic DNA from *Listeria monocytogenes*, *Escherichia coli*, *Salmonella enteritidis*, and *Staphylococcus aureus* were collected.

For sensitivity evaluation, a synthetic DNA fragment of the *T. gondii* B1 gene was utilized as the amplification template. Ten-fold serial dilutions were prepared, covering a concentration range of 100 nM to 10 fM, and 2 μL of each dilution was used for assessment. Additionally, *L. monocytogenes* genomic DNA was subjected to gradient dilution (500 pg/μL to 50 fg/μL) to evaluate the sensitivity of the proposed method.

### 2.7. Detection of Artificially Spiked Samples

To evaluate the performance of the established method for sample detection, we prepared 30 artificially spiked samples by adding target DNA into beef homogenate to simulate the sample matrix (not real-world samples). *T. gondii* samples: A 10 g portion of beef sample was minced and mixed with 90 mL of sterile saline solution (0.9% NaCl), followed by homogenization at medium speed for 15 min. The mixture was centrifuged at 500 r/min for 30 s to remove large food particles. After nucleic acid extraction, a *T. gondii* B1 gene fragment was added at a final concentration of 100 fM. *L. monocytogenes* samples: A 10 g portion of beef sample was minced and mixed with 90 mL of sterile saline solution (0.9% NaCl), followed by homogenization at medium speed for 15 min. To 900 μL of homogenate, *Listeria monocytogenes* genomic DNA at concentrations ranging from 50 pg/μL to 0.5 fg/μL was added and mixed thoroughly by vortexing. The spiked samples were centrifuged at 500 r/min for 30 s to remove large food particles. The supernatant was subjected to nucleic acid extraction via a DNA extraction kit (Yeasen Biotechnology, Shanghai, China) and subsequently detected using the Cascade-CRISPR assay.

### 2.8. Statistical Analysis

All graphical representations were generated using OriginPro 9.0, and statistical analyses were performed with SPSS Statistics 26.0. For fluorescence detection assays, three independent replicate experiments (n = 3) were conducted. Statistical comparisons were made using independent samples *t*-test, with a two-tailed *p*-value < 0.05 considered statistically significant.

## 3. Results

### 3.1. One-Tube Amplification-Free CRISPR/Cas12a Detection Workflow

**Figure 1:**
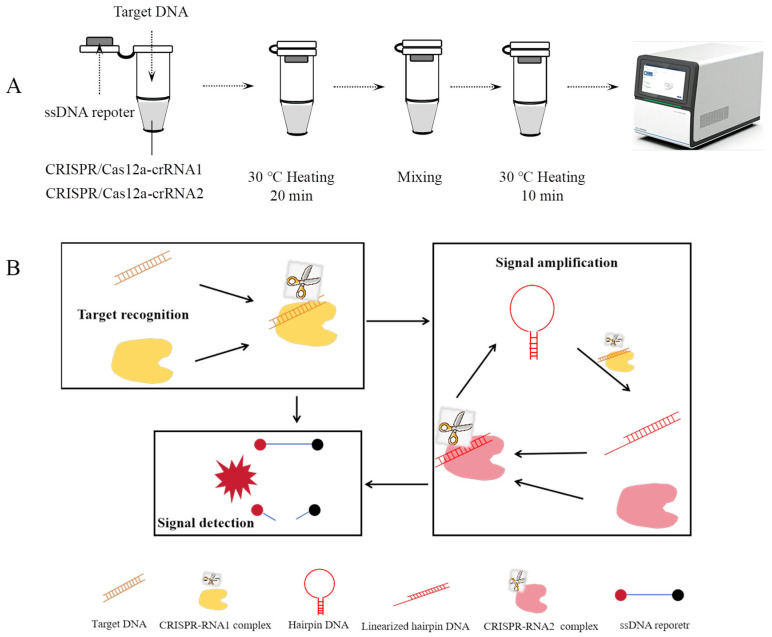
Amplification-free CRISPR-Cas12a detection. (**A**) Workflow of the amplification-free CRISPR-Cas12a assay. (**B**) Schematic diagram of the amplification-free CRISPR-Cas12a detection.

The amplification-free CRISPR/Cas12a detection process is illustrated in Figure 1A,B. This assay consists of three key steps: target recognition, signal amplification, and signal detection. When the target DNA fragment is present in the reaction system, the trans-cleavage activity of the RNP1 complex is activated. RNP1 cleaves the loop region of the hairpin DNA, converting it into linearized DNA. Subsequently, the linearized hairpin DNA activates the trans-cleavage activity of the RNP2 complex, which further cleaves additional hairpin DNA molecules, generating more linearized hairpin DNA and establishing a cascading amplification cycle. Simultaneously, the activated RNP1 and RNP2 complexes cleave single-stranded reporter DNA present in the system. The reporter DNA is labeled with a FAM fluorophore at one end and a BHQ1 quencher at the other. Upon cleavage, the fluorophore is separated from the quencher, resulting in a detectable fluorescence signal.

### 3.2. Feasibility Analysis of the Amplification-Free Detection System

**Figure 2:**
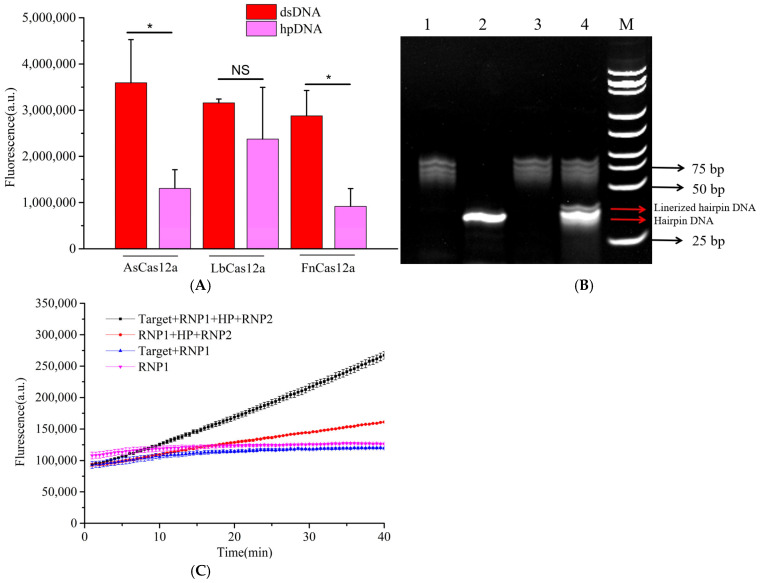
Feasibility analysis of the amplification-free detection system. (**A**) Screening of different Cas12a proteins; (**B**) verification of trans-cleavage activity on hairpin DNA: Lane 1: RNP1, Lane 2: 8T-HP, Lane 3: RNP1 + target, Lane 4: RNP1 + target + 8T-HP, M: DNA ladder; (**C**) validation of linearized hairpin DNA activation efficacy. *t*-test *p* < 0.05 (*), no significant difference (NS). In the chart, bars represent the mean average value, and the error bar represents the standard deviation.

To establish an amplification-free detection system using hairpin DNA, we first evaluated the inhibitory effect of hairpin DNA on the trans-cleavage activity of CRISPR/Cas12a proteins. As shown in Figure 2A, we tested three commonly used Cas12a proteins: FnCas12a, LbCas12a, and AsCas12a. The results demonstrated that hairpin DNA exhibited varying degrees of inhibition on the trans-cleavage activity of all three Cas12a proteins, with the weakest inhibition observed for LbCas12a. While FnCas12a and AsCas12a showed comparable inhibition effects, the AsCas12a detection system generated the strongest fluorescent signal when double-stranded DNA (dsDNA) was used as the target. Therefore, AsCas12a was selected for subsequent experiments.

Next, we assessed the cleavage efficiency of AsCas12a on hairpin DNA. In Figure 2B, comparison between lanes 2 and 4 revealed that after adding target DNA, the CRISPR/Cas12a-crRNA1 complex cleaved the hairpin DNA into two bands: an upper band corresponding to linearized hairpin DNA and a lower band representing intact hairpin DNA. This result confirmed partial cleavage of hairpin DNA into linear DNA.

We then evaluated the activation effect of linearized hairpin DNA on AsCas12a. The CRISPR/Cas12a-crRNA1 complex, targeting the *T. gondii* B1 gene, was used to cleave hairpin DNA. After the reaction, heat treatment at 85 °C for 10 min was performed to inactivate the CRISPR/Cas12a-crRNA1 complex. Upon cooling to room temperature, the hairpin DNA regained its double-stranded structure. Subsequently, the CRISPR/Cas12a-crRNA2 complex and single-stranded DNA (ssDNA) reporter probe were added to the system to measure fluorescence intensity. As illustrated in Figure 2C, when the CRISPR/Cas12a-crRNA2 complex (targeting linearized hairpin DNA) was introduced into the inactivated CRISPR/Cas12a-crRNA1 system, the reaction mixture containing linearized hairpin DNA produced a stronger fluorescent signal than that containing only intact hairpin DNA. This finding demonstrated that linearized hairpin DNA could effectively activate the trans-cleavage activity of AsCas12a. Collectively, these results confirm the feasibility of establishing an amplification-free CRISPR detection system using hairpin DNA.

### 3.3. Optimization of Amplification-Free Detection System

**Figure 3:**
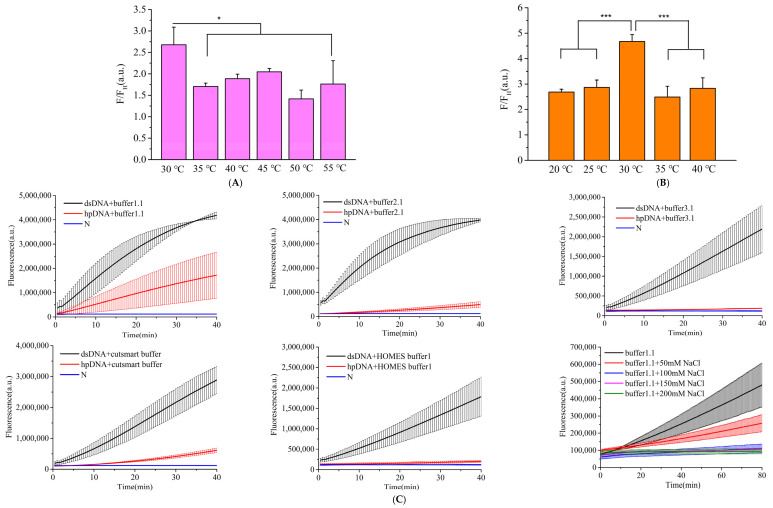

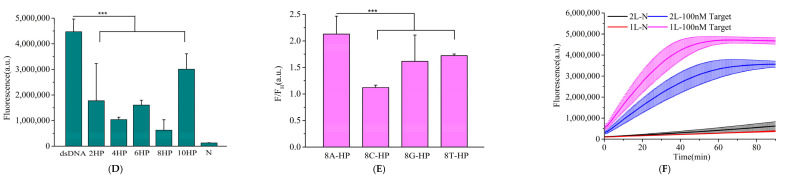
Optimization of the amplification-free detection system. (**A**) Temperature screening for CRISPR/Cas12a-crRNA1-mediated cleavage of hairpin DNA, (**B**) temperature screening for CRISPR/Cas12a-crRNA2-mediated cleavage of ssDNA reporter, (**C**) optimization of reaction buffer conditions, (**D**) screening of hairpin DNA loop length, (**E**) evaluation of hairpin DNA loop base composition, (**F**) optimization of hairpin DNA loop quantity. *t*-test *p* < 0.05 (*), *p* < 0.001 (***). In the chart, bars represent the mean average value, and the error bar represents the standard deviation.

Given that reaction temperature can influence the stability of hairpin DNA structures, we evaluated the trans-cleavage efficiency of the CRISPR/Cas12a-crRNA1 complex on hairpin DNA at 30 °C, 35 °C, 40 °C, 45 °C, 50 °C, and 55 °C. Under identical conditions, the fluorescence signal generated by linearized double-stranded DNA was divided by that produced by hairpin DNA as the evaluation metric. As shown in Figure 3A, the highest fold increase (2.6-fold) was observed at 30 °C, indicating optimal performance at this temperature. Subsequently, we assessed the cleavage efficiency of the CRISPR/Cas12a-crRNA2 complex on single-stranded reporter DNA at 20 °C, 25 °C, 30 °C, 35 °C, and 40 °C (Figure 3B). While the trans-cleavage activity of CRISPR/Cas12a-crRNA2 on single-stranded reporter DNA increased with rising temperature, the background signal also gradually intensified. A maximum fold change of 4.6 was observed at 30 °C, prompting its selection as the preferred reaction temperature.

Different reaction buffers can also affect the cleavage activity of the Cas12a protein. We evaluated 10× NEB buffer 1.1, 10× NEB buffer 2.1, 10× NEB buffer 3.1, 10× NEB CutSmart buffer, and 10× HOMLES buffer. Over time, the inhibitory effects of 10× NEB buffer 1.1, 10× NEB buffer 2.1, and 10× NEB CutSmart buffer gradually weakened, whereas 10× NEB buffer 3.1 and 10× HOMLES buffer demonstrated superior and comparable suppression efficacy (Figure 3C). Comparative analysis of buffer compositions revealed that the primary difference among 10× NEB buffer 1.1, 10× NEB buffer 2.1, and 10× NEB buffer 3.1 lay in NaCl concentration. By supplementing buffer 1.1 with varying NaCl concentrations, we observed a progressive reduction in background signal with increasing NaCl levels, suggesting that elevated NaCl concentrations effectively suppress background noise.

The composition of hairpin DNA also influences the cleavage efficiency of the Cas12a protein. We examined the inhibitory effects of hairpin DNAs with different loop lengths (2 bp, 4 bp, 6 bp, 8 bp, and 10 bp) on Cas12a at 35 °C for 60 min. The 8 bp loop length exhibited the strongest suppression (Figure 3D). Additionally, we evaluated the impact of different base compositions in the loop region on Cas12a cleavage, using the ratio of fluorescence signals from linearized hairpin DNA to hairpin DNA as the metric. As illustrated in Figure 3E, hairpin DNA with an adenine loop demonstrated the most favorable performance. The number of loops in the hairpin structure may also affect cleavage efficiency. Comparative analysis of hairpin DNAs with one versus two loops revealed that the single-loop variant generated stronger fluorescence signals and superior inhibitory effects (Figure 3F). Unexpectedly, we observed that biotin-modified hairpin DNA exhibited inferior inhibitory effects (Figure S2).

Ultimately, the optimized detection system conditions were determined as follows: reaction buffer (10× NEB buffer 3.1), AsCas12a as the detection protein, 30 °C for the CRISPR/Cas12a-crRNA1 detection system, 8 bp-adenine loop hairpin DNA as the signal amplifier, and 30 °C for the CRISPR/Cas12a-crRNA2 detection system.

### 3.4. Evaluation of Detection Sensitivity and Specificity

**Figure 4:**
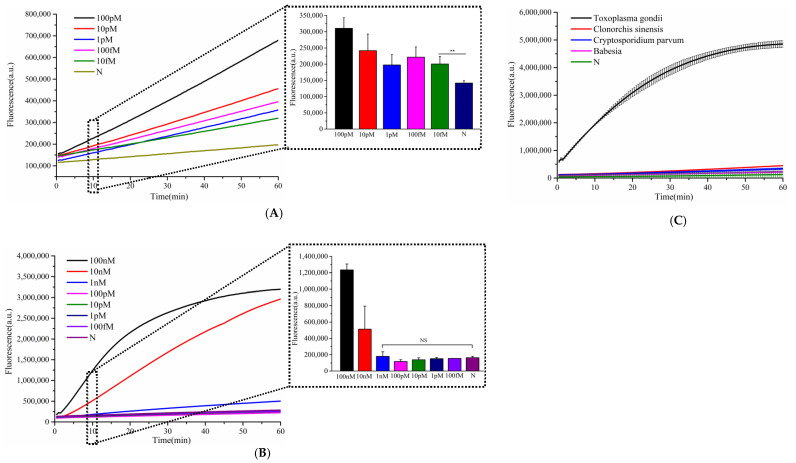
Evaluation of specificity and sensitivity for Toxoplasma gondii detection. (**A**) Sensitivity analysis of Cascade-CRISPR-based *T. gondii* detection, (**B**) sensitivity assessment of single Cas12a-mediated *T. gondii* detection, (**C**) specificity testing of Cascade-CRISPR system for *T. gondii* identification. In the chart, bars represent the mean average value, and the error bar represents the standard deviation. *t*-test *p* < 0.01 (**), no significant difference (NS).

To assess the specificity of the established detection system, we tested four distinct parasitic species. As shown in Figure 4A, only *T. gondii* generated a strong fluorescence signal, while non-*T. gondii* parasites exhibited no significant signal variation, confirming the high specificity of the assay. For sensitivity evaluation, we used a synthetic DNA fragment of the *T. gondii* B1 gene as the target. Figure 4B demonstrates that the system could detect concentrations as low as 10 fM, whereas single Cas12a detection methods only achieved a limit of detection of 1 nM—indicating a 10^5^-fold improvement in sensitivity. Notably, this method enabled the detection of *T. gondii* B1 gene fragments at 10 fM within just 30 min (20 min for target recognition and signal amplification, 10 min for detection).

**Figure 5:**
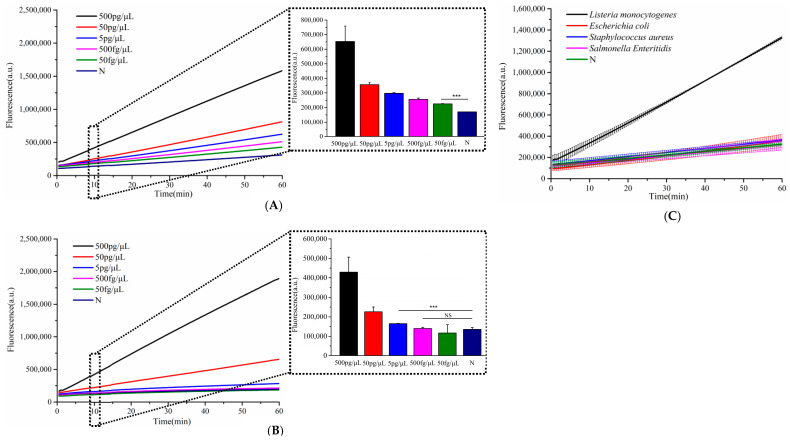
Evaluation of specificity and sensitivity for *Listeria monocytogenes* detection. (**A**) Sensitivity analysis of Cascade-CRISPR-based *L. monocytogenes* detection, (**B**) sensitivity assessment of single Cas12a-mediated *L. monocytogenes* detection, (**C**) specificity testing of Cascade-CRISPR system for *L. monocytogenes* identification. *t*-test *p* < 0.001 (***), no significant difference (NS).

To further demonstrate the broad applicability of the designed hairpin DNA signal amplifier, we selected the *L. monocytogenes* hemolysin virulence gene (*hly*) as the target and designed the corresponding crRNA1 to evaluate the method’s sensitivity and specificity. As illustrated in Figure 5, only genomic DNA from *L. monocytogenes* triggered a significantly enhanced fluorescence signal, while non-target samples produced negligible background signals. This confirms the high specificity of the detection system for *L. monocytogenes.* The assay demonstrated exceptional sensitivity, detecting *L. monocytogenes* DNA at concentrations as low as 50 fg/μL—a 100-fold improvement over single CRISPR-based methods, which had a detection limit of 5 pg/μL. Notably, this ultra-sensitive detection was achieved within 30 min, highlighting the rapidity and efficiency of the Cascade-CRISPR approach.

### 3.5. Detection of Artificially Spiked Samples

**Figure 6:**
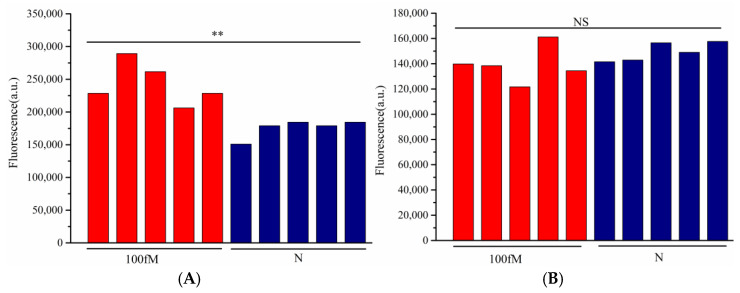
Results of 100 fM DNA detection in beef samples, detection based on Cascade-CRISPR (**A**), detection based on single Cas12a (**B**), *p* < 0.01 (**), no significant difference (NS).

The detection was performed on artificially contaminated raw beef samples, with quantitative fluorescence PCR (qPCR) serving as the control. As shown in Table 1, qPCR detected 10 positive samples out of 20, while Cascade-CRISPR also identified 10 positives. In contrast, the single Cas12a method only detected six positive samples. Notably, all samples missed by the single Cas12a method exhibited relatively low target concentrations. Consistent with these findings, the *T. gondii* detection results demonstrated that the single Cas12a method failed to detect nucleic acids at concentrations as low as 100 fM, whereas Cascade-CRISPR maintained detectable sensitivity at this level (Figure 6).

## 4. Discussion

In recent years, the increasing demand for rapid testing has driven significant advancements in molecular point-of-care testing (POCT) technologies. Within the field of foodborne pathogen detection, methodological evolution has progressed from conventional culture-based methods and classical PCR to various isothermal amplification techniques, including recombinase polymerase amplification (RPA), loop-mediated isothermal amplification (LAMP), and rolling circle amplification (RCA) [25,26,27]. However, practical implementation has revealed limitations: Isothermal amplification either requires complex reaction systems (e.g., RPA necessitates three distinct enzymes) or involves sophisticated primer design (e.g., LAMP requires 4–6 primers). Moreover, the constant temperature conditions predispose these methods to false-positive results [28], thereby restricting their widespread adoption. This technological gap has spurred interest in CRISPR-based detection systems. The integration of CRISPR with isothermal amplification addresses both the false-positive issues inherent to isothermal methods and the limited sensitivity of CRISPR to low-abundance targets. However, this combined approach increases operational complexity and elevates costs, highlighting the need for amplification-free CRISPR detection strategies.

Several innovative systems have emerged: Shi et al. [16] developed the CONAN system using fluorophore-labeled ssDNA/RNA hybrids as signal amplifiers, achieving 1 aM sensitivity within 20 min. Deng et al. [29] employed circular DNA amplifiers in the AutoCAR system (15–60 min for 1 aM detection). Sun et al. [30] created the CALSA system with LNA-modified ssDNA amplifiers (50 fM sensitivity in 1 h). Lim et al. [17] utilized biotinylated hairpin DNA for 1 aM detection in 10 min. While these studies demonstrated single-tube, isothermal detection using a solitary CRISPR protein, their signal amplifiers faced challenges in synthesis difficulty, high cost, and elevated background signals.

This study establishes a novel amplification-free CRISPR-based detection technology utilizing hairpin DNA as the signal amplifier and *AsCas12a* protein. The hairpin DNA amplifiers can be readily prepared through simple synthesis followed by annealing, offering significant advantages in both simplicity of production and cost-effectiveness. Elevated NaCl concentrations provided additional background suppression. While Zhou et al. [31] achieved femtomolar-level detection (83 fM) using hairpin DNA amplifiers with *LbCas12a*, their system exhibited substantial background interference. Previous studies have reported that elevated NaCl concentrations attenuate the trans-cleavage activity of Cas12a proteins [32]. In our system, the inherently weak activation of Cas12a’s trans-cleavage by hairpin DNA, combined with this salt-mediated suppression, synergistically reduced non-specific background signals. Unexpectedly, we observed that biotin modification actually increased background signals, indicating that this strategy lacks universal applicability. In conclusion, this study demonstrates a single-tube, amplification-free nucleic acid detection system utilizing hairpin DNA and AsCas12a protein. The hairpin DNA-based signal amplifier offers facile synthesis and low production cost, achieving femtomolar (fM) sensitivity within 30 min. Foodborne bacterial poisoning incidents require high timeliness in detection, making on-site rapid testing particularly crucial. The method developed in this study offers fast detection without the need for large-scale equipment, demonstrating excellent potential for field applications.

Although the Cascade-CRISPR detection method offers significant advantages over existing technologies, several aspects of this study require further optimization. For instance, while the background signal observed in our assay was relatively low, it remained substantially higher than that of the negative controls. This limitation suggests that structural modifications to the hairpin DNA probes may be necessary to improve assay specificity. Additionally, our study observed that nucleic acids rapidly extracted using automated extraction systems exhibit strong inhibition in CRISPR-based assays, while their impact on qPCR detection was minimal. These findings highlight the need to improve the impurity tolerance of CRISPR detection systems. It should be noted that this detection system was validated only with artificially simulated samples. For practical applications, factors such as sampling time and nucleic acid extraction efficiency may delay detection, necessitating further optimization in subsequent studies.

## 5. Conclusions

This study established an amplification-free detection system based on the AsCas12a protein by utilizing hairpin DNA. Through optimization of hairpin DNA composition, reaction buffer, and reaction temperature, the system achieved detection of DNA at the femtomolar (fM) level. By adjusting the NaCl concentration in the reaction buffer, the background signal of the reaction was effectively reduced. The high specificity and sensitivity of this method were demonstrated through the detection of *Toxoplasma gondii* and *Listeria monocytogenes*, with a short detection time of only 30 min. Notably, when detecting different targets, only RNP1 needed to be replaced, while RNP2 remained unchanged, indicating the universal applicability of the hairpin DNA amplifier in this system. Artificial contamination experiments further confirmed the feasibility of this method for detecting foodborne pathogens in real-world samples. In summary, this study developed a simple and practical amplification-free CRISPR detection method, which holds great potential for the rapid identification of foodborne pathogens.

## Figures and Tables

**Table 1:** Results of DNA detection in beef samples based on qPCR, single Cas12a, and Cascade-CRISPR.

Sample Number	DNA Concentration	Detection of *L. monocytogenes*		
		Single Cas12a	Cascade-CRISPR	qPCR
1	50 pg/μL	+	+	+
2	50 pg/μL	+	+	+
3	50 pg/μL	+	+	+
4	500 fg/μL	-	+	+
5	500 fg/μL	-	+	+
6	5 pg/μL	+	+	+
7	5 pg/μL	+	+	+
8	5 pg/μL	+	+	+
9	50 fg/μL	-	+	+
10	50 fg/μL	-	+	+
11	5 fg/μL	-	-	-
12	5 fg/μL	-	-	-
13	5 fg/μL	-	-	-
14	0.5 fg/μL	-	-	-
15	0.5 fg/μL	-	-	-
16	0	-	-	-
17	0	-	-	-
18	0	-	-	-
19	0	-	-	-
20	0	-	-	-

“+” indicates positive, “-” indicates negative.

## Data Availability

The original contributions presented in this study are included in the article/Supplementary Material. Further inquiries can be directed to the corresponding author.

## Supplementary Materials

The following supporting information can be downloaded at: https://www.mdpi.com/article/10.3390/microorganisms13081896/s1, Figure S1: Melting curves of hairpin DNA Act-H-8p-A, Act-H-2Loop-N, and Act-H-2Loop-Bio. Figure S2: Verification of trans-cleavage activation by biotin-labeled versus unlabeled hairpin DNAs on AsCas12a protein. Table S1. Oligonucleotide sequences utilized in this study.

## References

[1] Gootenberg J.S., Abudayyeh O.O., Lee J.W., Essletzbichler P., Dy A.J., Joung J., Verdine V., Donghia N., Daringer N.M., Freije C.A. (2017). Nucleic acid detection with CRISPR-Cas13a/C2c2. Science.

[2] Li S.Y., Cheng Q.X., Wang J.M., Li X.Y., Zhang Z.L., Gao S., Cao R.B., Zhao G.P., Wang J. (2018). CRISPR-Cas12a-assisted nucleic acid detection. Cell Discov..

[3] Harrington L.B., Burstein D., Chen J.S., Paez-Espino D., Ma E., Witte I.P., Cofsky J.C., Kyrpides N.C., Banfield J.F., Doudna J.A. (2018). Programmed DNA destruction by miniature CRISPR-Cas14 enzymes. Science.

[4] Chen J., Chen Y., Huang L., Lin X., Chen H., Xiang W., Liu L. (2025). Trans-nuclease activity of Cas9 activated by DNA or RNA target binding. Nat. Biotechnol..

[5] Xu H., Tang H., Li R., Xia Z., Yang W., Zhu Y., Liu Z., Lu G., Ni S., Shen J. (2022). A New Method Based on LAMP-CRISPR-Cas12a-Lateral Flow Immunochromatographic Strip for Detection. Infect. Drug Resist..

[6] Chen H., Zhang H., Guo J., Meng X., Yao M., He L., Nie X., Xu H., Liu C., Sun J. (2025). Rapid detection of feline parvovirus using RAA-CRISPR/Cas12a-based lateral flow strip and fluorescence. Front. Microbiol..

[7] Wang J., Zhu X., Yin D., Cai C., Liu H., Yang Y., Guo Z., Yin L., Shen X., Dai Y. (2023). Rapid and Easy-Read Porcine Circovirus Type 4 Detection with CRISPR-Cas13a-Based Lateral Flow Strip. Microorganisms.

[8] Shen J., Chen Z., Xie R., Li J., Liu C., He Y., Ma X., Yang H., Xie Z. (2023). CRISPR/Cas12a-Assisted isothermal amplification for rapid and specific diagnosis of respiratory virus on an microfluidic platform. Biosens. Bioelectron..

[9] Xu T., Cao F., Dai T., Liu T. (2024). RPA-CRISPR/Cas12a-Mediated Isothermal Amplification for Rapid Detection of *Phytopythium helicoides*. Plant Dis..

[10] Xu D., Zeng H., Wu W., Liu H., Wang J. (2023). Isothermal Amplification and CRISPR/Cas12a-System-Based Assay for Rapid, Sensitive and Visual Detection of *Staphylococcus aureus*. Foods.

[11] Lu S., Tong X., Han Y., Zhang K., Zhang Y., Chen Q., Duan J., Lei X., Huang M., Qiu Y. (2022). Fast and sensitive detection of SARS-CoV-2 RNA using suboptimal protospacer adjacent motifs for Cas12a. Nat. Biomed. Eng..

[12] Broughton J.P., Deng X., Yu G., Fasching C.L., Servellita V., Singh J., Miao X., Streithorst J.A., Granados A., Sotomayor-Gonzalez A. (2020). CRISPR-Cas12-based detection of SARS-CoV-2. Nat. Biotechnol..

[13] Ding X., Yin K., Li Z., Lalla R.V., Ballesteros E., Sfeir M.M., Liu C. (2020). Ultrasensitive and visual detection of SARS-CoV-2 using all-in-one dual CRISPR-Cas12a assay. Nat. Commun..

[14] Ye X., Wu H., Liu J., Xiang J., Feng Y., Liu Q. (2024). One-pot diagnostic methods based on CRISPR/Cas and Argonaute nucleases: Strategies and perspectives. Trends Biotechnol..

[15] Sha Y., Huang R., Huang M., Yue H., Shan Y., Hu J., Xing D. (2021). Cascade CRISPR/cas enables amplification-free microRNA sensing with fM-sensitivity and single-base-specificity. Chem. Commun..

[16] Shi K., Xie S., Tian R., Wang S., Lu Q., Gao D., Lei C., Zhu H., Nie Z. (2021). A CRISPR-Cas autocatalysis-driven feedback amplification network for supersensitive DNA diagnostics. Sci. Adv..

[17] Lim J., Van A.B., Koprowski K., Wester M., Valera E., Bashir R. (2025). Amplification-free, OR-gated CRISPR-Cascade reaction for pathogen detection in blood samples. Proc. Natl. Acad. Sci. USA.

[18] Hide G. (2016). Role of vertical transmission of *Toxoplasma gondii* in prevalence of infection. Expert. Rev. Anti Infect. Ther..

[19] Pereira K.S., Franco R.M., Leal D.A. (2010). Transmission of toxoplasmosis (*Toxoplasma gondii*) by foods. Adv. Food Nutr. Res..

[20] Dian S., Ganiem A.R., Ekawardhani S. (2023). Cerebral toxoplasmosis in HIV-infected patients: A review. Pathog. Glob. Health.

[21] Farber J.M., Peterkin P.I. (1991). *Listeria monocytogenes*, a food-borne pathogen. Microbiol. Rev..

[22] Bintsis T. (2017). Foodborne pathogens. AIMS Microbiol..

[23] Jeung J.H., Han H., Lee C.Y., Ahn J.K. (2023). CRISPR/Cas12a Collateral Cleavage Activity for Sensitive 3′-5′ Exonuclease Assay. Biosensors.

[24] Li L., Li S., Wu N., Wu J., Wang G., Zhao G., Wang J. (2019). HOLMESv2: A CRISPR-Cas12b-Assisted Platform for Nucleic Acid Detection and DNA Methylation Quantitation. ACS Synth. Biol..

[25] Aladhadh M. (2023). A Review of Modern Methods for the Detection of Foodborne Pathogens. Microorganisms.

[26] Bai W., Chen J., Chen D., Zhu Y., Hu K., Lin X., Chen J., Song D. (2024). Sensitive and rapid detection of three foodborne pathogens in meat by recombinase polymerase amplification with lateral flow dipstick (RPA-LFD). Int. J. Food Microbiol..

[27] Ndraha N., Lin H.Y., Wang C.Y., Hsiao H.I., Lin H.J. (2023). Rapid detection methods for foodborne pathogens based on nucleic acid amplification: Recent advances, remaining challenges, and possible opportunities. Food Chem..

[28] Li X., Zhang X., Shi X., Shi H., Wang Z., Peng C. (2022). Review in isothermal amplification technology in food microbiological detection. Food Sci. Biotechnol..

[29] Deng F., Li Y., Yang B., Sang R., Deng W., Kansara M., Lin F., Thavaneswaran S., Thomas D.M., Goldys E.M. (2024). Topological barrier to Cas12a activation by circular DNA nanostructures facilitates autocatalysis and transforms DNA/RNA sensing. Nat. Commun..

[30] Sun K., Pu L., Chen C., Chen M., Li K., Li X., Li H., Geng J. (2024). An autocatalytic CRISPR-Cas amplification effect propelled by the LNA-modified split activators for DNA sensing. Nucleic Acids Res..

[31] Zhou Z., Lau C.H., Wang J., Guo R., Tong S., Li J., Dong W., Huang Z., Wang T., Huang X. (2024). Rapid and Amplification-free Nucleic Acid Detection with DNA Substrate-Mediated Autocatalysis of CRISPR/Cas12a. ACS Omega.

[32] Hwang I., Song Y.H., Lee S. (2025). Enhanced trans-cleavage activity using CRISPR-Cas12a variant designed to reduce steric inhibition by cis-cleavage products. Biosens. Bioelectron..

