# Supplementary material for: Cascade CRISPR/cas Enables More Sensitive Detection of Toxoplasma gondii and Listeria monocytogenes than Single CRISPR/cas
Source: Microorganisms. 2025 Aug 14;13(8):1896. doi: 10.3390/microorganisms13081896 (PMC12388507; doi:10.3390/microorganisms13081896)
Supplement: Supplementary file 1 [file microorganisms-13-01896-s001.zip › microorganisms-3766812-supplementary.pdf]

Supplementary Information for
Cascade CRISPR/cas Enables More Sensitive Detection of *Toxoplasma gondii* and *Listeria monocytogenes* than Single CRISPR/cas

Table S1. Oligonucleotide sequences utilized in this study.

Name	Sequence	Application
GT-crRNA	UAAUUUCUACUGUUGUAGAUCUCUCUUCACUGUCACGUAC	crRNA for the B1 gene
Act-crRNA	UAAUUUCUACUCUUGUAGAUUUCCG-CAAUACUCCCCCAGGU	crRNA for Act DNA
Act-ssDNA	ACC TGG GGG AGT ATT GCG GAG GAA GGT	single-stranded Act DNA
Act-ssDNA-C	ACCTTCCTCCGCAATACTCCCCCAGGT	Complementary strand of Act DNA
T7-crRNA-F	GAAATTAATACGACTCACTATAGGG	In vitro transcription of crRNA targeting the hly gene of *L. monocytogenes*
T7-LM2-R2	CAGGGAGAACATCTGGTTGAATCTACAACAG-TAGAAATTCCCTATAGTGAGTCGTATTAATTTC	
ssDNA	5′FAM-TCTACTCTC-3′BHQ1	Reporter probe
Act-H-2p	GGA ACC TGG GGG AGT ATT GCG GAG GAATTTTC CTC CGC AAT ACT CCC CCA GGT TCC	Screening of hairpin DNA loop length
Act-H-4p	GGA ACC TGG GGG AGT ATT GCG GAG GAATTTTTTC CTC CGC AAT ACT CCC CCA GGT TCC	
Act-H-6p	GGA ACC TGG GGG AGT ATT GCG GAG GAATTTTTTTTC CTC CGC AAT ACT CCC CCAGGT TCC	
Act-H-8p	GGA ACC TGG GGG AGT ATT GCG GAG GAATTTTTTTTTTC CTC CGC AAT ACT CCC CCAGGT TCC	
Act-H-10p	GGA ACC TGG GGG AGT ATT GCG GAG GAATTTTTTTTTT TTCC TCC GCA ATA CTC CCCCAG GTT CC	
Act-H-8p-C	GGA ACC TGG GGG AGT ATT GCG GAG GAACCCCCCCCTTC CTC CGC AAT ACT CCC CCAGGT TCC	Screening of hairpin loop compositions
Act-H-8p-A	GGA ACC TGG GGG AGT ATT GCG GAG GAAAAAAAAAATTC CTC CGC AAT ACT CCC CCAGGT TCC	
Act-H-8p-G	GGA ACC TGG GGG AGT ATT GCG GAG GAAGGGGGGGGTTC CTC CGC AAT ACT CCC CCAGGT TCC	
GT-95DNA-T	GGCGGACCTCTCTTGTCTCGAATACACGAACGA-GATCTGCTGGATCTCTTCCCTTGAATGCGATGTCGTAC-GTGACAGTGAAGAGAGGAAACAGG	B1 gene fragment
Act-H-2Loop-Bio	Bio-TGGA ACC TGG GGG AGT ATT GCG GAG GATTTATTT TCC TCC GCA ATACTTATTTC CCC CAG GTTCCA	Screening of labeled hairpin loops
Act-H-2Loop-N	TGGA ACC TGG GGG AGT ATT GCG GAG GATTTATTT TCC TCC GCA ATACTTATTTC CCC CAG GTTCCA	

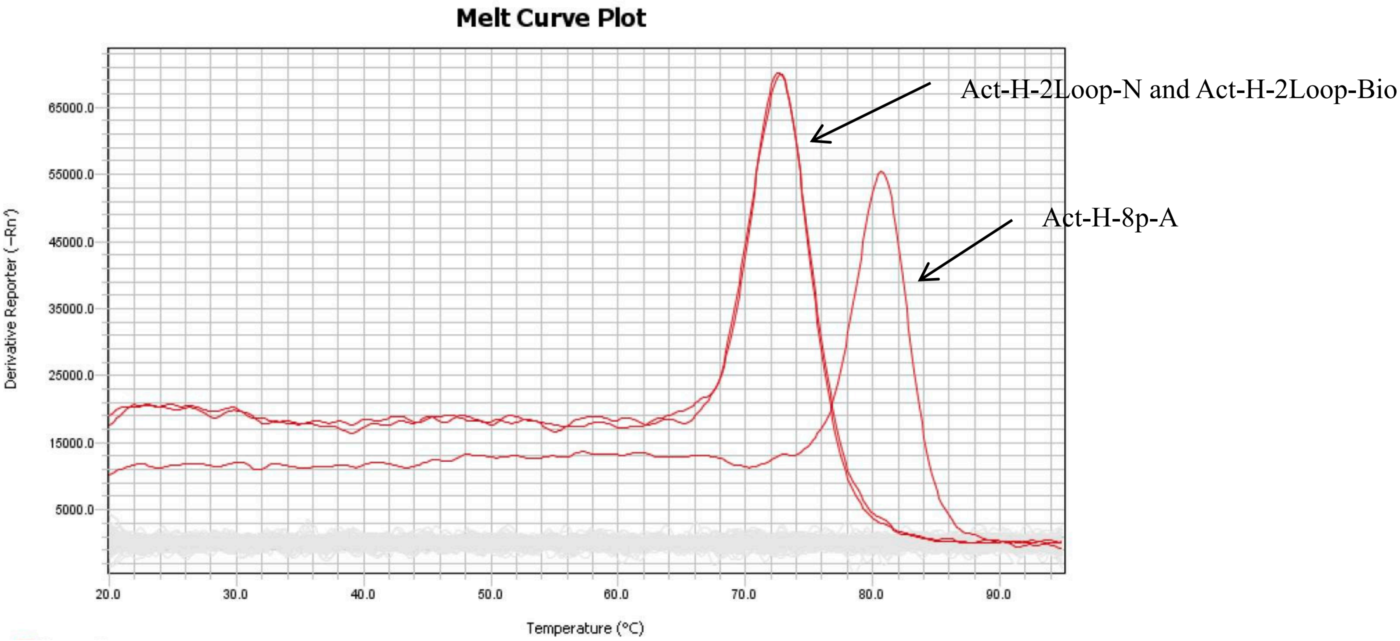

Figure S1. Melting curves of hairpin DNA Act-H-8p-A, Act-H-2Loop-N, and Act-H-2Loop-Bio.

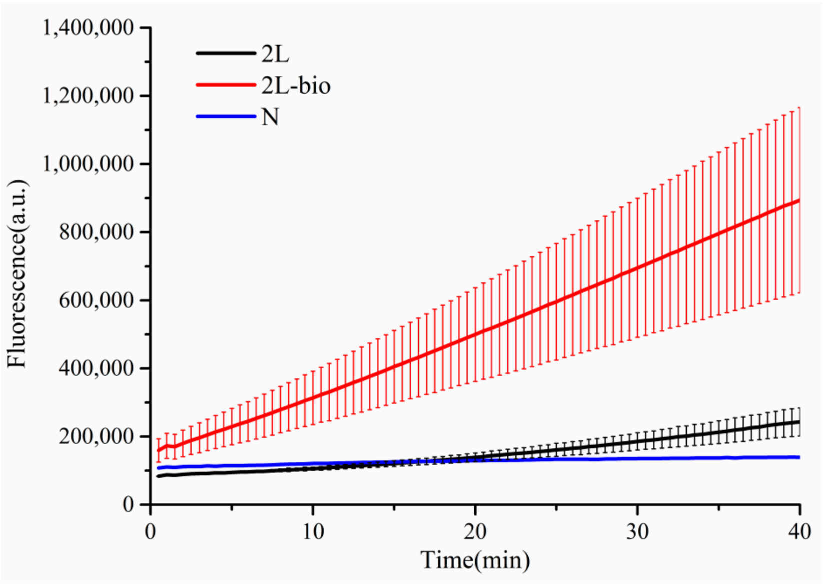

Figure S2. Verification of trans-cleavage activation by biotin-labeled versus unlabeled hairpin DNAs on AsCas12a protein.
